# Spatiotemporal dynamics in a diffusive Leslie–Gower model: Turing instability and bifurcations with prey Allee effect and predator fear effect

**DOI:** 10.1016/j.idm.2026.10.004

**Published:** 2026-10-26

**Authors:** Ye Han, Wanqin Wu, Xuewen Tan, Xinzhi Liu, Wenjie Li

**Affiliations:** a School of Mathematics and Computer Science, Yunnan Minzu University, Kunming, Yunnan, 650500, China; b Department of Applied Mathematics, University of Waterloo, Waterloo, N2L 3G1, Canada; c Institute for Differential Equation Theory and Applications, Faculty of Science, Kunming University of Science and Technology, Kunming, Yunnan, 650500, China

**Keywords:** Leslie–Gower predator–prey, Strong Allee effect, Diffusive, Turing instability

## Abstract

This paper deals with a diffusive Leslie–Gower model with a strong Allee effect on the prey and a fear effect on the predator. We first consider the stability of positive constant solutions of the system in the absence of diffusion. Furthermore, we analyze the Turing instability phenomenon in positive constant solutions resulting from diffusion effects. Moreover, we investigate steady-state bifurcations using the Lyapunov–Schmidt reduction. The research establishes that diffusion can induce instability in these solutions. To validate these findings, we present numerical simulations, suggesting the model's potential to generate spatial patterns under specific conditions.

## 1 Introduction

In this paper, we consider a reaction–diffusion Leslie–Gower model with strong Allee effect on prey and fear effect on predator

(1.1) $$\left\{\begin{array}{ll} \frac{\partial v}{\partial t}-d_{v}\Delta v=rv\left(1-\frac{v}{K}\right)\left(v-m\right)-\frac{qv\omega }{1+k\omega }, & t>0,x\in \Omega ,\\ \frac{\partial \omega }{\partial t}-d_{\omega }\Delta \omega =\frac{s\omega }{1+k\omega }\left(1-\frac{\omega }{nv}\right), & t>0,x\in \Omega , \end{array}\right)$$

where *v* represents the density of the prey population, *ω* denotes the density of the predator population, *r* stands for the intrinsic growth rate of the prey, *s* indicates the intrinsic growth rate of the predator, *K* is the environmental carrying capacity of the prey, *m* denotes the Allee effect threshold of the prey, *q* represents the maximum per capita consumption rate of the predator on the prey, *n* is the proportional coefficient between the predator's environmental carrying capacity and prey density, *k* quantifies the fear effect parameter on the predator, and the initial conditions for system (1.1) satisfy *v*(*t*) ≥ 0 and *ω*(*t*) ≥ 0, with all parameters *r*, *K*, *m*, *q*, *k*, *s*, *n* being positive constants. *d*_*v*_ is prey diffusion coefficient, *d*_*ω*_ is predator diffusion coefficient. Here $\Omega \subset R^{N}$ (*N* ≥ 1) is a bounded domain with a smooth boundary *∂*Ω.

The basic idea of system (1.1) originates from Liu et al. (2022), where the original system is an ordinary differential equation (ODE) system. As far as we know, the diffusion term can describe the phenomena of population migration, invasion and isolation in real habitats, such as fish migration and insect diffusion. The formation of diffusion-driven spatial patterns can explain the plate-like distribution in nature, such as the zonation of fish in coral reefs and the aggregation of locusts. In particular, diffusion analysis can identify the risk of local extinction, locate the habitats of endangered species, and guide practices such as habitat corridor design and isolation zones for invasive species. It is known that diffusive Turing instability is significant. For example, when the diffusion rates of the predator and the prey are significantly different, the system breaks the homogeneous steady state through Turing bifurcation and spontaneously forms ordered spatial structures, such as periodic high-low-density bands. Furthermore, the Turing instability condition can quantify the critical diffusion ratio. When environmental disturbances cause system parameters to exceed the threshold, the homogeneous state may suddenly change to a speckle pattern state, indicating the risk of local extinction or the outbreak of invasive species. Such phenomena have been extensively studied, including Hopf bifurcation (Chen et al., 2023; Dai & Sun, 2021; Ding et al., 2025; Tao & Wang, 2025), Turing instability (Wang et al., 2024) and pattern formation (An & Jiang, 2018; Li et al., 2025a; Zhang et al., 2019; Zhao & Shen, 2025), etc. Recently, Li and his collaborators have systematically investigated the threshold dynamics and bifurcation behaviors in various reaction-diffusion epidemic models, including those with saturation incidence (Li et al., 2025b), media impact (Li et al., 2024a), heterogeneous environments (Li et al., 2023a), and discontinuous incidence (Li et al., 2025c). Their recent work also extends to predator-prey systems, exploring Turing-Hopf bifurcation under Allee and fear effects (Li et al., 2026a). Complementing these studies, other researchers have examined nonlinear dynamics in epidemic models incorporating information and saturated treatment (Das et al., 2021), as well as host-vector interactions with saturated incidence (Han et al., 2026). In parallel, pattern formation and bifurcation analysis have been advanced in predator-prey systems with fractional-order dynamics (Wu et al., 2025), alternative prey (Lei et al., 2024), anti-predation behavior (Yang et al., 2025), constant predator release (Zhou et al., 2022), ratio-dependent functional response with Allee effect (Xu & He, 2022), and fear effects on eco-epidemiological dynamics (Zhang, 2024). Discrete epidemic models have also been developed to incorporate media impact, resource constraints, and switching strategies (Qin et al., 2025). Recently, Li et al. (2026b) investigated Turing instability and pattern formation in a diffusive predator–prey system with opportunistic predators and a weak Allee effect. However, their model does not incorporate the fear effect on the prey, and the Allee effect considered therein is of the weak type. In contrast, the present study focuses on a Leslie–Gower predator–prey model with a strong Allee effect on the prey and a fear effect on the predator. Moreover, besides the diffusion-driven Turing instability, we further investigate the steady-state bifurcations via the Lyapunov–Schmidt reduction. However, to the best of our knowledge, there are few theoretical results on Turing instability of positive solutions for a diffusive Leslie–Gower model with strong Allee effect on prey and fear effect on predator. The central focus of this study is to analyze the impact of diffusion and supplementary diffusive effects on the Turing instability and existence of positive constant solutions for model (1.1). Resolving these questions involves considerable complexity.

The structure of this work is organized as follows: Section 2 explores the stability and existence of positive constant solutions when diffusion is absent in model (1.1). Subsequently, Section 3 delves into the diffusion-induced Turing instability of these solutions. Section 4 is devoted to the analysis of steady-state bifurcations. Section 5 presents numerical simulations to illustrate and support the theoretical findings. Finally, Section 6 concludes this paper with a summary of the main results and a discussion of possible future research directions.

## 2 Stability of positive constant solutions

In the absence of diffusion, model (1.1) reduces to

(2.1) $$\left\{\begin{array}{ll} \frac{dv}{dt}=rv\left(1-\frac{v}{K}\right)\left(v-m\right)-\frac{qv\omega }{1+k\omega }, & t>0,\\ \frac{d\omega }{dt}=\frac{s\omega }{1+k\omega }\left(1-\frac{\omega }{nv}\right), & t>0. \end{array}\right)$$

It is evident that (2.1) possesses the trivial solution (0, 0) and semi-trivial solutions (*K*, 0) and (*m*, 0). In what follows, we examine the existence of positive constant solutions for model (2.1). Assume that (*v*∗, *ω*∗) represents a positive constant solution of (2.1). Then, it follows that *ω*∗ = *nv*∗, where *v*∗ is the positive root of the cubic equation *a*_1_*v*^3^ + *b*_1_*v*^2^ + *c*_1_*v* + *d*_1_ = 0, with coefficients defined as $a_{1}=kn,b_{1}=1-Kkn-kmn,c_{1}=Kkmn-K-m+\frac{Kqn}{r},d_{1}=Km$. We consider the real root of the following equation:

$$knv^{3}+\left(1-Kkn-kmn\right)v^{2}+\left(Kkmn-K-m+\frac{Kqn}{r}\right)v+Km=0.$$

Let

$$G\left(v\right)=knv^{3}+\left(1-Kkn-kmn\right)v^{2}+\left(Kkmn-K-m+\frac{Kqn}{r}\right)v+Km,$$

$$g\left(v\right)=\frac{dG\left(v\right)}{dv}=3knv^{2}+2\left(1-Kkn-kmn\right)v+\left(Kkmn-K-m+\frac{Kqn}{r}\right).$$

Define

$$A={\left(1-Kkn-kmn\right)}^{2}-3kn\left(Kkmn-K-m+\frac{Kqn}{r}\right),B=\left(1-Kkn-kmn\right)\left(Kkmn-K-m+\frac{Kqn}{r}\right)-9knKm,C={\left(Kkmn-K-m+\frac{Kqn}{r}\right)}^{2}-3\left(1-Kkn-kmn\right)Km,\Delta =B^{2}-4AC.$$

Obviously, *G*(0) = *Km* > 0. System (2.1) has at most two positive equilibrium points.

Using Descartes’ Sign Rule and the Shengjin Formula, we can readily derive the existence of positive equilibria as follows.

Lemma 2.1 *For system* (2.1)*, the following conclusions hold.*

1. *If*
  $$Kkmn+\frac{Kqn}{r}<K+m$$
  *or if*
  $$Kkmn+\frac{Kqn}{r}>K+m\text{and}Kkn+kmn>1,$$
  *then the following conclusions hold*:
  (a) *If* Δ < 0*, there exist two positive equilibria* $E_{1}^{*}\left(v_{1}^{*},\omega_{1}^{*}\right)$ *and* $E_{2}^{*}\left(v_{2}^{*},\omega_{2}^{*}\right)$*;*
  (b) *If* Δ = 0*,*
    • *if* A>0*, there exists a unique positive equilibrium* *E*∗(*v*∗, *ω*∗)*;*
    • *if* A=0*, there exists no positive equilibrium;*
  (c) *If* Δ > 0*, there exists no positive equilibrium;*
2. *If* $Kkmn+\frac{Kqn}{r}\geq K+m$*,* *Kkn* + *kmn* ≤ 1*, there exists no positive equilibrium.*

This section is dedicated to analyzing the stability of the positive constant solutions $\left(v_{i}^{*},\omega_{i}^{*}\right)$ *i* = 1, 2. The Jacobian matrix of (2.1) at $\left(v_{i}^{*},\omega_{i}^{*}\right)$, *i* = 1, 2 is

$$J_{0}\left(v_{i}^{*},\omega_{i}^{*}\right)=\left(\begin{array}{ll} L_{1}\left(v_{i}^{*},\omega_{i}^{*}\right) & L_{2}\left(v_{i}^{*},\omega_{i}^{*}\right)\\ L_{3}\left(v_{i}^{*},\omega_{i}^{*}\right) & L_{4}\left(v_{i}^{*},\omega_{i}^{*}\right) \end{array}\right),$$

where

$$L_{1}\left(v_{i}^{*},\omega_{i}^{*}\right)=-\frac{3r}{K}{v_{i}^{*}}^{2}+2\left(\frac{mr}{K}+r\right)v_{i}^{*}-mr-\frac{q\omega_{i}^{*}}{1+k\omega_{i}^{*}},L_{2}\left(v_{i}^{*},\omega_{i}^{*}\right)=-\frac{qv_{i}^{*}}{{\left(1+k\omega_{i}^{*}\right)}^{2}}<0,L_{3}\left(v_{i}^{*},\omega_{i}^{*}\right)=\frac{sn}{1+k\omega_{i}^{*}},L_{4}\left(v_{i}^{*},\omega_{i}^{*}\right)=-\frac{s}{1+k\omega_{i}^{*}}<0.$$

Then the characteristic equation for $J_{0}\left(v_{i}^{*},\omega_{i}^{*}\right)$ is given by $u^{2}-\text{tr}\left(J_{0}\left(v_{i}^{*},\omega_{i}^{*}\right)\right)u+\det\left(J_{0}\left(v_{i}^{*},\omega_{i}^{*}\right)\right)=0$ with

$$\begin{array}{ll} \text{tr}\left(J_{0}\left(v_{i}^{*},\omega_{i}^{*}\right)\right)=L_{1}\left(v_{i}^{*},\omega_{i}^{*}\right)+L_{4}\left(v_{i}^{*},\omega_{i}^{*}\right)=-\frac{3r}{K}{v_{i}^{*}}^{2}+2\left(\frac{mr}{K}+r\right)v_{i}^{*}-mr-\frac{q\omega_{i}^{*}}{1+k\omega_{i}^{*}}+\left(-\frac{s}{1+k\omega_{i}^{*}}\right), & \; \end{array}$$

$$\begin{array}{ll} \det\left(J_{0}\left(v_{i}^{*},\omega_{i}^{*}\right)\right)=L_{1}\left(v_{i}^{*},\omega_{i}^{*}\right)L_{4}\left(v_{i}^{*},\omega_{i}^{*}\right)-L_{2}\left(v_{i}^{*},\omega_{i}^{*}\right)L_{3}\left(v_{i}^{*},\omega_{i}^{*}\right) & \;\\ =\left(-\frac{3r}{K}{v_{i}^{*}}^{2}+2\left(\frac{mr}{K}+r\right)v_{i}^{*}-mr-\frac{q\omega_{i}^{*}}{1+k\omega_{i}^{*}}\right)\left(-\frac{s}{1+k\omega_{i}^{*}}\right)-\left(-\frac{qv_{i}^{*}}{{\left(1+k\omega_{i}^{*}\right)}^{2}}\right)\left(\frac{sn}{1+k\omega_{i}^{*}}\right) & \\ =\left(-\frac{3r}{K}{v_{i}^{*}}^{2}+2\left(\frac{mr}{K}+r\right)v_{i}^{*}-mr-\frac{q\omega_{i}^{*}}{1+k\omega_{i}^{*}}\right)\left(-\frac{s}{1+k\omega_{i}^{*}}\right)+\frac{sqnv_{i}^{*}}{{\left(1+k\omega_{i}^{*}\right)}^{3}}.\;\;\;\;\;\;\; & \end{array}$$

Clearly, $\det\left(J_{0}\left(v_{i}^{*},\omega_{i}^{*}\right)\right)>0$ when $2\left(\frac{mr}{K}+r\right)v_{i}^{*}<\frac{3r}{K}{v_{i}^{*}}^{2}+mr+\frac{q\omega_{i}^{*}}{1+k\omega_{i}^{*}}$. Denote $s_{0}^{i}=L_{1}\left(v_{i}^{*},\omega_{i}^{*}\right)\left(1+k\omega_{i}^{*}\right),i=1,2.$ Consequently, the subsequent conclusion may be derived.

Lemma 2.2 *Assuming that* $2\left(\frac{mr}{K}+r\right)v_{i}^{*}<\frac{3r}{K}{v_{i}^{*}}^{2}+mr+\frac{q\omega_{i}^{*}}{1+k\omega_{i}^{*}}$ *holds, the solution* $\left(v_{i}^{*},\omega_{i}^{*}\right)$ *exhibits local asymptotic stability when* $s>s_{0}^{i}$ *and is unstable when* $s_{0}^{i}>s$*,* *i* = 1, 2.

## 3 Turing instability

Evidently, the positive constant solutions $\left(v_{i}^{*},\omega_{i}^{*}\right)$, where *i* = 1, 2, of system (2.1) simultaneously function as constant positive solutions for Eq. (1.1). In this section, our focus is on investigating the diffusion-driven Turing instability of these solutions $\left(v_{i}^{*},\omega_{i}^{*}\right)$, *i* = 1, 2. To proceed, we first assume that $\left(v_{i}^{*},\omega_{i}^{*}\right)$ in (2.1) is locally asymptotically stable (LAS), which requires the fulfillment of the condition **(H1)**.

**(H1)** $\;s>s_{0}^{i}\;\text{and}\;2\left(\frac{mr}{K}+r\right)v_{i}^{*}<\frac{3r}{K}{v_{i}^{*}}^{2}+mr+\frac{q\omega_{i}^{*}}{1+k\omega_{i}^{*}},i=1,2$.

The linearization of Eq. (1.1) at point $\left(v_{i}^{*},\omega_{i}^{*}\right)$ is given by

$$\frac{d}{dt}\left(\begin{array}{l} v\\ \omega \end{array}\right)=J\left(v_{i}^{*},\omega_{i}^{*}\right)\left(\begin{array}{l} v\\ \omega \end{array}\right)$$

with

$$J\left(v_{i}^{*},\omega_{i}^{*}\right)=\left(\begin{array}{ll} d_{v}\Delta +L_{1}\left(v_{i}^{*},\omega_{i}^{*}\right) & L_{2}\left(v_{i}^{*},\omega_{i}^{*}\right)\\ L_{3}\left(v_{i}^{*},\omega_{i}^{*}\right) & d_{\omega }\Delta +L_{4}\left(v_{i}^{*},\omega_{i}^{*}\right) \end{array}\right)$$

and $L_{j}\left(v_{i}^{*},\omega_{i}^{*}\right),j=1,\ldots ,4$ being the same as that of defined in Section 2. Define $J_{j}\left(v_{i}^{*},\omega_{i}^{*}\right)$ as

(3.1) $$J_{j}\left(v_{i}^{*},\omega_{i}^{*}\right)=\left(\begin{array}{ll} -d_{v}u_{j}+L_{1}\left(v_{i}^{*},\omega_{i}^{*}\right) & L_{2}\left(v_{i}^{*},\omega_{i}^{*}\right)\\ L_{3}\left(v_{i}^{*},\omega_{i}^{*}\right) & -d_{\omega }u_{j}+L_{4}\left(v_{i}^{*},\omega_{i}^{*}\right) \end{array}\right)$$

with *u*_*j*_ representing the eigenvalue of the negative Laplacian operator −Δ on the domain Ω. Subsequently, the characteristic equation corresponding to (3.1) is

$$u^{2}-\text{tr}\left(J_{j}\left(v_{i}^{*},\omega_{i}^{*}\right)\right)u+\det\left(J_{j}\left(v_{i}^{*},\omega_{i}^{*}\right)\right)=0$$

with

$$\text{tr}\left(J_{j}\left(v_{i}^{*},\omega_{i}^{*}\right)\right)=-\left(d_{v}+d_{\omega }\right)u_{j}+\text{tr}\left(J_{0}\left(v_{i}^{*},\omega_{i}^{*}\right)\right),\det\left(J_{j}\left(v_{i}^{*},\omega_{i}^{*}\right)\right)=d_{v}d_{\omega }u_{j}^{2}-\left(d_{v}L_{4}\left(v_{i}^{*},\omega_{i}^{*}\right)+d_{\omega }L_{1}\left(v_{i}^{*},\omega_{i}^{*}\right)\right)u_{j}+\det\left(J_{0}\left(v_{i}^{*},\omega_{i}^{*}\right)\right).$$

Under condition **(H1)**, $\text{tr}\left(J_{j}\left(v_{i}^{*},\omega_{i}^{*}\right)\right)<0$ for all *j* ≥ 0 and $\det\left(J_{0}\left(v_{i}^{*},\omega_{i}^{*}\right)\right)>0$. Additionally, if there exists some *j* > 0 such that $\det\left(J_{j}\left(v_{i}^{*},\omega_{i}^{*}\right)\right)<0$, the solution $\left(v_{i}^{*},\omega_{i}^{*}\right)$ of (1.1) becomes unstable, an instability clearly driven by diffusion (Turing instability). Given that $\det\left(J_{0}\left(v_{i}^{*},\omega_{i}^{*}\right)\right)>0$, the necessary and sufficient conditions for Turing instability to occur are:

**(H2)**$\;d_{v}L_{4}\left(v_{i}^{*},\omega_{i}^{*}\right)+d_{\omega }L_{1}\left(v_{i}^{*},\omega_{i}^{*}\right)>0$,

**(H3)**$\;{\left(d_{v}L_{4}\left(v_{i}^{*},\omega_{i}^{*}\right)+d_{\omega }L_{1}\left(v_{i}^{*},\omega_{i}^{*}\right)\right)}^{2}-4d_{v}d_{\omega }\det\left(J_{0}\left(v_{i}^{*},\omega_{i}^{*}\right)\right)>0$.

In the subsequent analysis, we aim to identify the conditions under which both **(H1)** and **(H2)** are satisfied, thereby establishing the Turing instability for the solutions $\left(v_{i}^{*},\omega_{i}^{*}\right)$, *i* = 1, 2 of model (1.1). The findings are presented as follows.

Theorem 3.1 *Assume that the condition* ***(H1)*** *is satisfied, and let* $s_{1}^{i},i=1,2$ *be as defined in equation* (3.3)*. Under these circumstances, the subsequent assertions are valid:*

(I) *If* $L_{1}\left(v_{i}^{*},\omega_{i}^{*}\right)<0$*, Turing instability cannot occur.*
(II) *Assuming that* $L_{1}\left(v_{i}^{*},\omega_{i}^{*}\right)>0$ *is satisfied, the following outcomes can be derived:*
  (i) *If* *d*_*v*_ ≥ *d*_*ω*_*, Turing instability cannot occur.*
  (ii) *If* *d*_*v*_ < *d*_*ω*_ *and* $s_{1}^{i}\leq s_{0}^{i}$*, Turing instability cannot occur.*
  (iii) *If* *d*_*v*_ < *d*_*ω*_ *and* $s_{1}^{i}>s_{0}^{i}$*, then Turing instability occurs for* $s\in \left(s_{0}^{i},s_{1}^{i}\right)$*.*

Proof (*I*) *Conclusion* (*I*) *of* Theorem 3.1 *is straightforward because if* $L_{1}\left(v_{i}^{*},\omega_{i}^{*}\right)<0$, **(*H*2)** *fails to be satisfied*.

(II) Let $L_{1}\left(v_{i}^{*},\omega_{i}^{*}\right)>0$. If *d*_*v*_ ≥ *d*_*ω*_, then $d_{v}L_{4}\left(v_{i}^{*},\omega_{i}^{*}\right)+d_{\omega }L_{1}\left(v_{i}^{*},\omega_{i}^{*}\right)\leq d_{v}\left(L_{4}\left(v_{i}^{*},\omega_{i}^{*}\right)+L_{1}\left(v_{i}^{*},\omega_{i}^{*}\right)\right)\leq 0$, which runs counter to **(H2)**. Therefore, no Turing instability occurs.

If *d*_*v*_ < *d*_*ω*_, condition **(H2)** is tantamount to $0<s<\frac{d_{\omega }L_{1}\left(v_{i}^{*},\omega_{i}^{*}\right)\left(1+k\omega_{i}^{*}\right)}{d_{v}}≜s_{*}^{i}$ and (**H3**) is tantamount to

(3.2) $$\begin{array}{ll} f\left(s\right)≜{d_{v}}^{2}L_{4}^{2}\left(v_{i}^{*},\omega_{i}^{*}\right)+{d_{\omega }}^{2}L_{1}^{2}\left(v_{i}^{*},\omega_{i}^{*}\right)-2d_{v}d_{\omega }L_{4}\left(v_{i}^{*},\omega_{i}^{*}\right)L_{1}\left(v_{i}^{*},\omega_{i}^{*}\right)+4d_{v}d_{\omega }L_{3}\left(v_{i}^{*},\omega_{i}^{*}\right)L_{2}(v_{i}^{*},\omega_{i}^{*}) & \;\\ ≜\frac{{d_{v}}^{2}s^{2}}{{\left(1+k\omega_{i}^{*}\right)}^{2}}+2d_{v}d_{\omega }\left[\frac{L_{1}\left(v_{i}^{*},\omega_{i}^{*}\right)}{1+k\omega_{i}^{*}}+2\frac{nL_{2}\left(v_{i}^{*},\omega_{i}^{*}\right)}{1+k\omega_{i}^{*}}\right]s+{d_{\omega }}^{2}L_{1}^{2}\left(v_{i}^{*},\omega_{i}^{*}\right)>0. & \end{array}$$

Set

$$\begin{array}{ll} \Delta \left(v_{i}^{*},\omega_{i}^{*}\right)=4{d_{v}}^{2}{d_{\omega }}^{2}{\left[\frac{L_{1}\left(v_{i}^{*},\omega_{i}^{*}\right)}{1+k\omega_{i}^{*}}+2\frac{nL_{2}\left(v_{i}^{*},\omega_{i}^{*}\right)}{1+k\omega_{i}^{*}}\right]}^{2}-4{d_{v}}^{2}{d_{\omega }}^{2}\frac{L_{1}^{2}\left(v_{i}^{*},\omega_{i}^{*}\right)}{{\left(1+k\omega_{i}^{*}\right)}^{2}} & \;\\ =16{d_{v}}^{2}{d_{\omega }}^{2}\frac{nL_{2}\left(v_{i}^{*},\omega_{i}^{*}\right)}{1+k\omega_{i}^{*}}\left[\frac{nL_{2}\left(v_{i}^{*},\omega_{i}^{*}\right)}{1+k\omega_{i}^{*}}+\frac{L_{1}\left(v_{i}^{*},\omega_{i}^{*}\right)}{1+k\omega_{i}^{*}}\right] & \\ =16{d_{v}}^{2}{d_{\omega }}^{2}\frac{nL_{2}\left(v_{i}^{*},\omega_{i}^{*}\right)}{{\left(1+k\omega_{i}^{*}\right)}^{2}}\frac{\det\left(J_{0}\left(v_{i}^{*},\omega_{i}^{*}\right)\right)}{L_{4}\left(v_{i}^{*},\omega_{i}^{*}\right)}. & \; \end{array}$$

Given that $\det\left(J_{0}\left(v_{i}^{*},\omega_{i}^{*}\right)\right)>0$ under $L_{2}\left(v_{i}^{*},\omega_{i}^{*}\right)<0$ and under the premise of assumption **(H1)**, it follows that Δ > 0. Consequently, *f*(*s*) = 0 possesses two positive roots $s_{1}^{i}$ and $s_{2}^{i}$, as expressed by

(3.3) $$s_{1}^{i}=\frac{-b_{2}-\sqrt{\Delta \left(v_{i}^{*},\omega_{i}^{*}\right)}}{2a_{2}}>0\;\text{and}\;s_{2}^{i}=\frac{-b_{2}+\sqrt{\Delta \left(v_{i}^{*},\omega_{i}^{*}\right)}}{2a_{2}}>0,$$

respectively, where $a_{2}=\frac{{d_{v}}^{2}}{{\left(1+k\omega_{i}^{*}\right)}^{2}},$ $b_{2}=2d_{v}d_{\omega }\left[\frac{L_{1}\left(v_{i}^{*},\omega_{i}^{*}\right)}{1+k\omega_{i}^{*}}+2\frac{nL_{2}\left(v_{i}^{*},\omega_{i}^{*}\right)}{1+k\omega_{i}^{*}}\right],$ and it is straightforward to deduce that *f*(*s*) > 0 when $0<s<s_{1}^{i}$ or $s>s_{2}^{i}$. By substituting $s_{*}^{i}$ into Equation (3.2), we obtain

$$f\left(s_{*}^{i}\right)=4{d_{\omega }}^{2}L_{1}\left(v_{i}^{*},\omega_{i}^{*}\right)\left[L_{1}\left(v_{i}^{*},\omega_{i}^{*}\right)+nL_{2}\left(v_{i}^{*},\omega_{i}^{*}\right)\right]<0,$$

this leads to the inequality $0<s_{1}^{i}<s_{*}^{i}<s_{2}^{i}$. As a result, both conditions **(H2)** and **(H3)** are satisfied when $0<s<s_{1}^{i}$, but fail to hold for $s\geq s_{1}^{i}$. Additionally, given *d*_*v*_ < *d*_*ω*_, condition **(H1)** is valid for $s>s_{0}^{i}$, and $s_{*}^{i}>s_{0}^{i}$. Consequently, Turing instability arises when $s_{0}^{i}<s<s_{1}^{i}$, while it cannot occur when $s_{1}^{i}\leq s_{0}^{i}$. The proofs of (ii) and (iii) are derived from this reasoning. □

Notice that Theorem 3.1 indicates that for model (1.1), when the intrinsic growth rate *s* of the predator falls within the interval $\left(s_{0}^{i},s_{1}^{i}\right)$, spatial patterns may emerge near the positive constant solutions $\left(v_{i}^{*},\omega_{i}^{*}\right),i=1,2$.

## 4 Existence of nonconstant steady-state solutions

In this section, we mainly aim to investigate the existence of nonconstant steady-state solutions near a positive constant steady state $E_{i}=\left(v_{i}^{*},\omega_{i}^{*}\right)$ under assumption **(H1)**.

(4.1) $$\left\{\begin{array}{l} 0=d_{v}\Delta v+rv\left(1-\frac{v}{K}\right)\left(v-m\right)-\frac{qv\omega }{1+k\omega },\\ 0=d_{\omega }\Delta \omega +\frac{s\omega }{1+k\omega }\left(1-\frac{\omega }{nv}\right),\\ \partial_{\nu }v=\partial_{\nu }\omega =0,\;x\in \partial \Omega . \end{array}\right)$$

The first Fréchet derivative of system (4.1) at *E*_*i*_ can be given as follows:

(4.2) $$L_{s}≔\left[\begin{array}{ll} d_{v}\Delta +L_{1}\left(v_{i}^{*},\omega_{i}^{*}\right) & L_{2}\left(v_{i}^{*},\omega_{i}^{*}\right)\\ L_{3}\left(v_{i}^{*},\omega_{i}^{*}\right) & d_{\omega }\Delta +L_{4}\left(v_{i}^{*},\omega_{i}^{*}\right) \end{array}\right],$$

Moreover, the adjoint operator $L_{s}^{*}$ of *L*_*s*_ reads as follows:

$$L_{s}^{*}≔\left[\begin{array}{ll} d_{v}\Delta +L_{1}\left(v_{i}^{*},\omega_{i}^{*}\right) & L_{3}\left(v_{i}^{*},\omega_{i}^{*}\right)\\ L_{2}\left(v_{i}^{*},\omega_{i}^{*}\right) & d_{\omega }\Delta +L_{4}\left(v_{i}^{*},\omega_{i}^{*}\right) \end{array}\right],$$

To investigate the existence of nonconstant steady state solutions near the positive constant steady state $E_{i}=\left(v_{i}^{*},\omega_{i}^{*}\right)$, we choose the predators intrinsic growth rate *s* as a bifurcation parameter and present the following hypothesis: **(**${\text{H}}_{j_{0}}$**)** $s<s_{1}^{i}$, $\det J_{j_{0}}\left(u_{j_{0}},s^{0}\right)=0$ and $\det J_{k}\left(u_{k},s^{0}\right)\neq 0$ for all $k\in N_{0}\setminus \left\{j_{0}\right\}$.

From the condition **(**${\text{H}}_{j_{0}}$**)** and (4.2), we obtain

$$s^{0}=-\frac{\left(1+k\omega_{i}^{*}\right)\left(d_{v}d_{\omega }u_{j_{0}}^{2}-d_{\omega }u_{j_{0}}L_{1}\left(v_{i}^{*},\omega_{i}^{*}\right)\right)}{d_{v}u_{j_{0}}-L_{1}\left(v_{i}^{*},\omega_{i}^{*}\right)+n\frac{qv_{i}^{*}}{{\left(1+k\omega_{i}^{*}\right)}^{2}}}.\;$$

Let $K=\ker L_{s^{0}}$ and $K^{*}=\ker L_{s^{0}}^{*}$. Under hypothesis **(**${\text{H}}_{j_{0}}$**)**, both K and $K^{*}$ are one-dimensional and we can choose

$$P=\text{span}\left\{q_{j_{0}}\psi_{j_{0}}\right\},\;P^{*}=\text{span}\left\{p_{j_{0}}\psi_{j_{0}}\right\},$$

where

$$q_{j_{0}}=\left(\begin{array}{l} 1\\ \frac{d_{v}u_{j_{0}}-L_{1}\left(v_{i}^{*},\omega_{i}^{*}\right)}{L_{2}\left(v_{i}^{*},\omega_{i}^{*}\right)} \end{array}\right),\;p_{j_{0}}=\left(\begin{array}{l} d_{\omega }u_{j_{0}}-L_{4}\left(v_{i}^{*},\omega_{i}^{*}\right)\\ L_{2}\left(v_{i}^{*},\omega_{i}^{*}\right) \end{array}\right).$$

In addition, from the expressions of $q_{j_{0}}$ and $p_{j_{0}}$, we derive

$$q_{j_{0}}\cdot p_{j_{0}}=\left(d_{v}+d_{\omega }\right)u_{j_{0}}-L_{1}\left(v_{i}^{*},\omega_{i}^{*}\right)-L_{4}\left(v_{i}^{*},\omega_{i}^{*}\right).$$

Furthermore, we define

$$\kappa_{0}≔q_{j_{0}}\cdot L_{s^{0}}\;p_{j_{0}}.$$

A direct calculation shows that

$$\det J_{j_{0}}\left(u_{j_{0}},s\right)=d_{v}d_{\omega }\;u_{j_{0}}^{2}-\left(d_{v}L_{4}\left(v_{i}^{*},\omega_{i}^{*}\right)+d_{\omega }L_{1}\left(v_{i}^{*},\omega_{i}^{*}\right)\right)u_{j_{0}}+\det J_{0}\left(v_{i}^{*},\omega_{i}^{*}\right).$$

To study the bifurcating nonconstant steady states of system (1.1) near the positive equilibrium $E_{i}=\left(v_{i}^{*},\omega_{i}^{*}\right)$, where $G={\left(G_{1},G_{2}\right)}^{\text{T}}:X^{2}\times R\to Y^{2}$ is defined by

$$\begin{array}{ll} G_{1}\left(v,\omega \right) & =d_{v}\Delta v+rv\left(1-\frac{v}{K}\right)\left(v-m\right)-\frac{qv\omega }{1+k\omega },\\ G_{2}\left(v,\omega \right) & =d_{\omega }\Delta \omega +\frac{s\omega }{1+k\omega }\left(1-\frac{\omega }{nv}\right), \end{array}$$

Let $E={\left(v,\omega \right)}^{⊤}\in X^{2}$ and s∈R. Then we are interested in nonconstant steady-state solutions of G(E,s)=0, when $E\to E_{i}\in X^{2}$ and $s\to s^{0}\in R$. For any

$$f={\left(f_{1},f_{2}\right)}^{⊤},\;g={\left(g_{1},g_{2}\right)}^{⊤},\;h={\left(h_{1},h_{2}\right)}^{⊤}\in K,$$

the Fréchet derivative forms of *d*^2^*F*_*z*_(**f**, **g**) and *d*^3^*F*_*z*_(**f**, **g**, **h**) are respectively

$$d^{2}F_{z}\left(f,g\right)=\left(\begin{array}{l} A_{1}\;f_{1}g_{1}+A_{2}\left(f_{1}g_{2}+f_{2}g_{1}\right)+A_{3}\;f_{2}g_{2}\\ A_{4}\;f_{1}g_{1}+A_{5}\left(f_{1}g_{2}+f_{2}g_{1}\right)+A_{6}\;f_{2}g_{2} \end{array}\right),$$

and

$$\begin{array}{l} d^{3}F_{z}\left(f,g,h\right)=\left(\begin{array}{l} B_{1}\;f_{1}g_{1}h_{1}+B_{2}\left(f_{1}g_{1}h_{2}+f_{1}g_{2}h_{1}+f_{2}g_{1}h_{1}\right)\\ \;+B_{3}\left(f_{1}g_{2}h_{2}+f_{2}g_{1}h_{2}+f_{2}g_{2}h_{1}\right)+B_{4}\;f_{2}g_{2}h_{2}\\ C_{1}\;f_{1}g_{1}h_{1}+C_{2}\left(f_{1}g_{1}h_{2}+f_{1}g_{2}h_{1}+f_{2}g_{1}h_{1}\right)\\ \;+C_{3}\left(f_{1}g_{2}h_{2}+f_{2}g_{1}h_{2}+f_{2}g_{2}h_{1}\right)+C_{4}\;f_{2}g_{2}h_{2} \end{array}\right). \end{array}$$

Here

$$\begin{array}{llll} A_{1} & =r\left[-\frac{6v_{i}^{⁎}}{K}+2\left(1+\frac{m}{K}\right)\right],\; & A_{2} & =-\;\frac{q}{{\left(1+knv_{i}^{⁎}\right)}^{2}},\;\\ A_{3} & =\frac{2qv_{i}^{⁎}k}{{\left(1+knv_{i}^{⁎}\right)}^{3}}, & A_{4} & =-\;\frac{2sn}{v_{i}^{⁎}\left(1+knv_{i}^{⁎}\right)},\;\\ A_{5} & =\frac{s\left(2+knv_{i}^{⁎}\right)}{v_{i}^{⁎}{\left(1+knv_{i}^{⁎}\right)}^{2}},\; & A_{6} & =-\frac{2s}{nv_{i}^{⁎}{\left(1+knv_{i}^{⁎}\right)}^{2}}, \end{array}$$

and

$$\begin{array}{llll} B_{1} & =-\;\frac{6r}{K},\; & B_{2} & =0,\;\\ B_{3} & =\frac{2qk}{{\left(1+knv_{i}^{⁎}\right)}^{3}}, & B_{4} & =-\;\frac{6qv_{i}^{⁎}k^{2}}{{\left(1+knv_{i}^{⁎}\right)}^{4}},\;\\ C_{1} & =\frac{6sn}{{v_{i}^{⁎}}^{2}\left(1+knv_{i}^{⁎}\right)},\; & C_{2} & =-\;\frac{2s\left(2+knv_{i}^{⁎}\right)}{{v_{i}^{⁎}}^{2}{\left(1+knv_{i}^{⁎}\right)}^{2}},\\ C_{3} & =\frac{2s}{n{v_{i}^{⁎}}^{2}{\left(1+knv_{i}^{⁎}\right)}^{3}},\; & C_{4} & =\frac{6sk}{nv_{i}^{⁎}{\left(1+knv_{i}^{⁎}\right)}^{3}}. \end{array}$$

One has the following decompositions

$$X^{2}=P⊕X_{j_{0}},\;Y^{2}=P^{*}⊕Y_{j_{0}},$$

and

$$X_{j_{0}}=\left\{\phi \in X^{2}\;\left|\;\langle \psi_{j_{0}},\;q_{j_{0}}\cdot \phi \rangle =0\right\},\;Y_{j_{0}}=\left\{\phi \in Y^{2}\;\right|\;\langle \psi_{j_{0}},\;p_{j_{0}}\cdot \phi \rangle =0\right\}.$$

Based on the analysis in (Cao et al., 2021; Li et al., 2022, 2023b, 2024b, 2026c), one knows that $L_{s}:X^{2}\to Y^{2}$ is a Fredholm operator of index zero and that the restriction

$${\left(L_{s}\right|}_{X_{j_{0}}}:X_{j_{0}}⟶Y_{j_{0}}$$

is invertible and has a bounded inverse. Let *N* and *I* − *N* be the projection operators from $Y^{2}$ onto $Y_{j_{0}}$ and *P*∗, respectively. Then, for any $\Phi \in Y^{2}$, one has

$$N\Phi =\Phi -\frac{\left\langle \psi_{j_{0}},\;q_{j_{0}}\cdot \Phi \right\rangle }{q_{j_{0}}\cdot p_{j_{0}}}\psi_{j_{0}}q_{j_{0}}.$$

Hence, the equation G(E,s)=0 is equivalent to the system

(4.3) NG(E,s)=0,(I−N)G(E,s)=0.

For every $E\in X^{2}$, there is a unique decomposition

$$E=E_{i}+Z\;\psi_{j_{0}}q_{j_{0}}+W,\;Z\in R,\;W\in X_{j_{0}}.$$

Hence, NG(E,s)=0 is equivalent to

$$NG\left(E_{i}+Z\psi_{j_{0}}q_{j_{0}}+W,\;s\right)=0.$$

Define a map

$$NG:K\times X_{j_{0}}\times R⟶Y_{j_{0}},$$

which satisfies the conditions of the implicit function theorem:

$$NG\left(E_{i},s\right)=0,\;NG_{W}\left(E_{i},s\right)=NL_{s}=L_{s}.$$

Therefore, one obtains a unique continuously differentiable map $W={\left(W_{1},W_{2}\right)}^{⊤}$, defined on (−*ɛ*, *ɛ*) × (*s*^0^ − *δ*, *s*^0^ + *δ*), where (−*ɛ*, *ɛ*) is an open neighborhood of the origin in R and (*s*^0^ − *δ*, *s*^0^ + *δ*) is an open neighborhood of *s*^0^ in R, which satisfies

$$\text{for all}\left(Z,s\right)\in \left(-\epsilon ,\epsilon \right)\times \left(s^{0}-\delta ,s^{0}+\delta \right),\;W\left(0,s\right)=0.$$

Substituting $E=E_{i}+Z\psi_{j_{0}}q_{j_{0}}+W\left(Z,s\right)$ into the first equation of (4.3), then

(4.4) $$\forall \;\left(Z,s\right)\in \left(-\epsilon ,\epsilon \right)\times \left(s^{0}-\delta ,s^{0}+\delta \right),\;NG\left(E_{i}+Z\psi_{j_{0}}q_{j_{0}}+W\left(Z,s\right),s\right)=0.$$

In addition, one has

$$\left\langle \psi_{j_{0}},\;q_{j_{0}}\cdot W\left(Z,s\right)\right\rangle \equiv 0.$$

Consequently, $\forall \;\left(Z,s\right)\in \left(-\epsilon ,\epsilon \right)\times \left(s^{0}-\delta ,s^{0}+\delta \right)$,

(4.5) $$G\left(E_{i}+Z\psi_{j_{0}}q_{j_{0}}+W\left(Z,s\right),\;s\right)\in K^{*}.$$

Substituting W=W(Z,s) into the second equation of (4.3), then

$$G\left(Z,s\right)\overset{\Delta }{=}\left(I-N\right)\;G\left(E_{i}+Z\psi_{j_{0}}q_{j_{0}}+W\left(Z,s\right),\;s\right)=0.$$

From (4.4), then

$$\begin{array}{ll} W\left(Z,s\right) & =\frac{1}{2}\;W_{2}Z^{2}+\frac{1}{6}\;W_{3}Z^{3}+\cdots \;,\\ W_{2} & =-{\left(L_{s^{0}}\right)}^{-1}d^{2}F_{z}\left(\psi_{j_{0}}q_{j_{0}},\;\psi_{j_{0}}q_{j_{0}}\right), \end{array}$$

where

$${\left(L_{s^{0}}\right)}^{-1}=\frac{1}{\det L_{s^{0}}}\left[\begin{array}{ll} d_{\omega }\Delta +L_{4}\left(v_{i}^{*},\omega_{i}^{*}\right) & -L_{2}\left(v_{i}^{*},\omega_{i}^{*}\right)\\ -L_{3}\left(v_{i}^{*},\omega_{i}^{*}\right) & d_{v}\Delta +L_{1}\left(v_{i}^{*},\omega_{i}^{*}\right) \end{array}\right].$$

Calculating the inner product of (4.5) with $q_{j_{0}}\psi_{j_{0}}$, we have

$$G^{*}\left(Z,s\right)=Z\;\left(\kappa_{0}+\frac{1}{2}\kappa_{1}Z+\frac{1}{6}\kappa_{2}Z^{2}\right)+o\left(|s-s^{0}|+|Z{|}^{3}\right),$$

where

$$\begin{array}{ll} \kappa_{1} & =\langle \psi_{j_{0}},\;q_{j_{0}}\cdot d^{2}F_{z}\left(\psi_{j_{0}}q_{j_{0}},\;\psi_{j_{0}}q_{j_{0}}\right)\rangle ,\\ \kappa_{2} & =\langle \psi_{j_{0}},\;q_{j_{0}}\cdot d^{3}F_{z}\left(\psi_{j_{0}}q_{j_{0}},\;\psi_{j_{0}}q_{j_{0}},\;\psi_{j_{0}}q_{j_{0}}\right)\rangle \\ & \;+3\langle \psi_{j_{0}},\;q_{j_{0}}\cdot d^{2}F_{z}\left(\psi_{j_{0}}q_{j_{0}},\;W_{ZZ}\left(0,s^{0}\right)\right)\rangle . \end{array}$$

If *κ*_1_ ≠ 0, then by the implicit function theorem there exist open neighbourhoods *ɛ* of 0 and *δ* of *s*^0^ in R, and a unique continuously differentiable map

s↦Z(s),

such that $G^{*}\left(Z\left(s\right),s\right)=0$. Moreover,

(4.6) $$Z\left(s\right)=-\frac{2\kappa_{0}}{\kappa_{1}}+o\left(\left|s-s^{0}\right|\right).$$

Hence, system (4.1) admits a nonconstant steady state

$$E_{s}=E_{i}+Z\left(s\right)\;q_{j_{0}}\psi_{j_{0}}+W\left(Z\left(s\right),s\right).$$

For convenience, for any *ɛ* > 0 we define the following intervals

$$I_{1}\left(s^{0},\epsilon \right)≔\left\{s\in \left(s^{0}-\epsilon ,s^{0}+\epsilon \right):\kappa_{0}>0\right\},$$

$$I_{2}\left(s^{0},\epsilon \right)≔\left\{s\in \left(s^{0}-\epsilon ,s^{0}+\epsilon \right):\kappa_{0}<0\right\}.$$

If *κ*_1_ = 0, *κ*_2_ < 0 (respectively >0), then there exist *ɛ* > 0 and two continuously differentiable mappings $Z^{\pm }$ from *I*_1_(*s*^0^, *ɛ*) (respectively, *I*_2_(*s*^0^, *ɛ*)) to R such that system (4.1) has two nontrivial solutions $E_{s}^{\pm }\in X^{2}$, where

$$E_{s}^{\pm }=E_{i}+Z^{\pm }\left(s\right)q_{j_{0}}\psi_{j_{0}}+W\left(Z^{\pm }\left(s\right),s\right),$$

when $s\to s^{0},E_{s}^{\pm }\to E_{i}$.

Theorem 4.1 *Assume that hypothesis* (${\text{H}}_{j_{0}}$) *holds.*

(i) *If* *κ*_1_ ≠ 0*, there exists a continuously differentiable map* s↦Z(s) *in* $\left(-\epsilon ,\epsilon \right)\subset R,\left(s^{0}-\delta ,s^{0}+\delta \right)\subset R$*, such that system* (4.1) *admits only one non-constant steady-state solution near* *E*_*i*_*, given by* $E_{s}=E_{i}+Z\left(s\right)q_{j_{0}}\psi_{j_{0}}+W\left(Z\left(s\right),s\right)$*. In addition* $\lim_{s\to s^{0}}E_{s}=E_{i}$*.*
(ii) *If* *κ*_1_ = 0, *κ*_2_ < 0 *(respectively* >0*), then there exist* *ɛ* > 0 *and two continuously differentiable mappings* $Z^{\pm }$ *from* *I*_1_(*s*^0^, *ɛ*) *(respectively,* *I*_2_(*s*^0^, *ɛ*)*) to* R *such that* (4.1) *has two nontrivial solutions* $E_{s}^{\pm }\in X^{2}$*, where*

$$E_{s}^{\pm }=E_{i}+Z^{\pm }\left(s\right)q_{j_{0}}\psi_{j_{0}}+W\left(Z^{\pm }\left(s\right),s\right),$$

*when* $s\to s^{0},E_{s}^{\pm }\to E_{i}$.

## 5 Numerical simulations

In this section, we have presented numerical simulations to support and extend our analytical results. Fixed parameters: *d*_*v*_ = 0.01, *d*_*ω*_ = 50, *r* = 0.15, *K* = 50.0, *m* = 0.15, *q* = 1.2, *k* = 0.1, *s* = 1.2, *n* = 0.1. The positive equilibrium is (*v*∗, *ω*∗) = (14.800, 1.480). Fig. 1 illustrates the evolution process of the population density distribution of the predator-prey model from time *t* = 1 to *t* = 8. At *t* = 1, *t* = 2, the pattern presents a relatively random and dispersed state, and the color distribution is relatively chaotic. At this time, the ecological variables are in a relatively disordered state, as shown in Fig. 1(a) and (b). At *t* = 4, *t* = 5, as time progresses, we can observe that the color distribution begins to show some aggregation trends. In some areas, the color gradually deepens and forms small aggregation areas. At this time, the distribution pattern begins to transform from disorder to order, see Fig. 1(c) and (d). At *t* = 6, *t* = 8 (Fig. 1(e) and (f)), obvious and large-scale color aggregation areas are formed in the picture, presenting relatively regular block or spot structures. At this time, ecological variables gradually form a stable and concentrated distribution pattern in space.

**Fig. 1:**
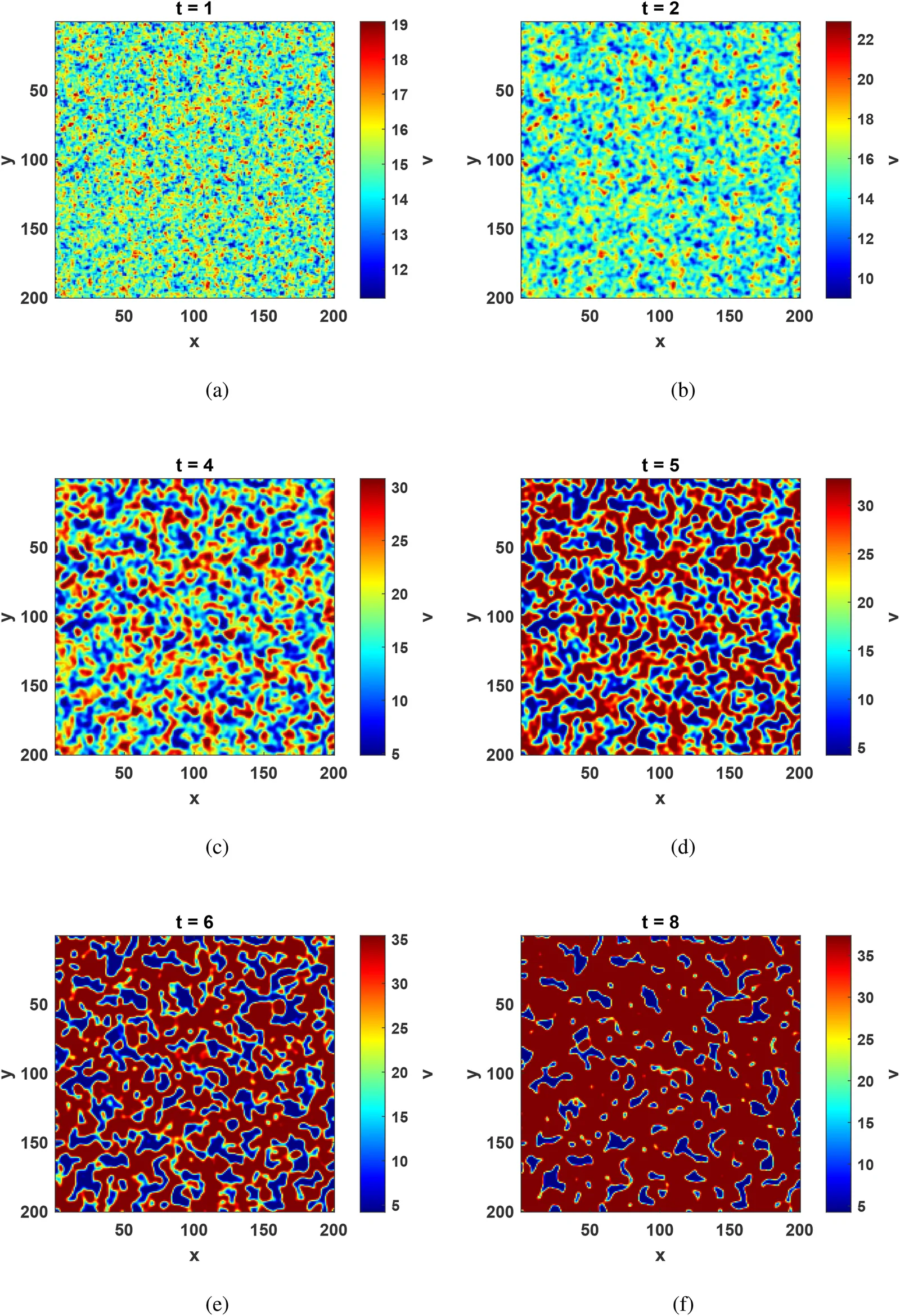
Two-dimensional numerical heat maps of the prey density *v* in model (1.1) with *d*_*v*_ = 0.01, *d*_*ω*_ = 50, *r* = 0.15, *K* = 50.0, *m* = 0.15, *q* = 1.2, *k* = 0.1, *s* = 1.2, *n* = 0.1.

We now investigate the pattern formation of the diffusive predator-prey model on the torus surface. To this end, keeping the values of the above parameters unchanged, by straightforward calculation, the positive equilibrium point (*v*∗, *ω*∗) = (14.800, 1.480) is obtained. Fig. 2 shows the distribution of population density on the torus surface at different times. At *t* = 1 and *t* = 2 (Fig. 2(a) and (b)), the color distribution on the torus surface is relatively chaotic at this time, without obvious aggregation or regular distribution characteristics. At *t* = 4 (Fig. 2(c)), compared with the previous moments, the density differences in some areas are more significant; From *t* = 5 to *t* = 8 (Fig. 2(d)–(f)), at this time, the color distribution shows a more obvious block aggregation, with a strong contrast between the red and blue areas. The distribution pattern changes from disorder to order. As time increases, the density values of specific areas change, and overall, it shows a relatively stable population density distribution pattern.

**Fig. 2:**
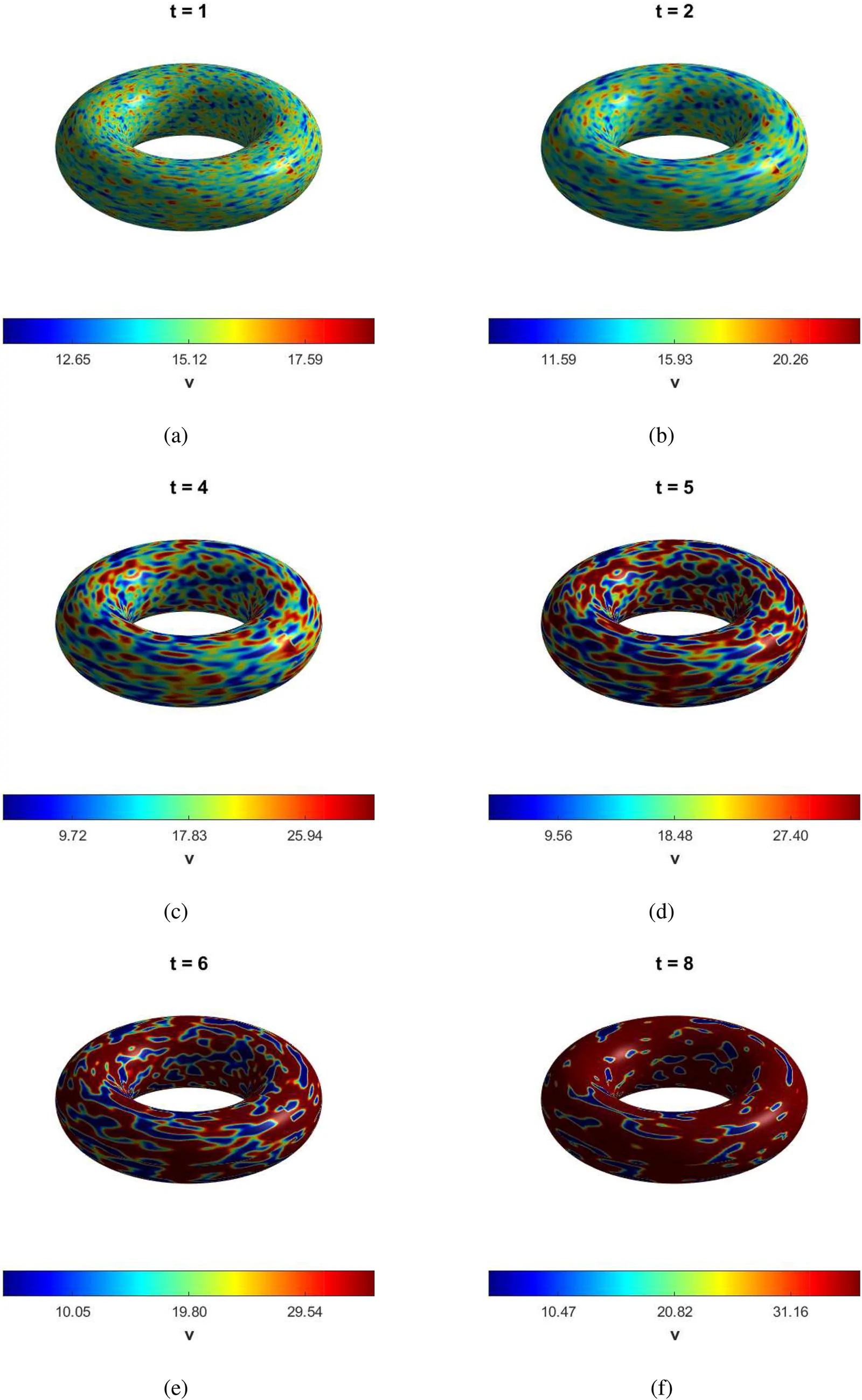
Three-dimensional numerical heat maps of the prey density *v* on the torus surface in model (1.1) with *d*_*v*_ = 0.01, *d*_*ω*_ = 50, *r* = 0.15, *K* = 50.0, *m* = 0.15, *q* = 1.2, *k* = 0.1, *s* = 1.2, *n* = 0.1.

Our research results help us better understand the predator-prey system and the interaction between predators and prey in the ecosystem.

## 6 Conclusion

This paper investigates a diffusive Leslie–Gower predator–prey model incorporating a strong Allee effect on the prey and a fear effect on the predator. We first establish the stability conditions for the positive constant steady states in the non-diffusive system. The existence of equilibria is characterized using Descartes' Rule of Signs and the Shengjin Formula, while the local asymptotic stability is determined by a threshold value associated with the predator's intrinsic growth rate. When spatial diffusion is introduced, we derive explicit critical conditions for Turing instability. These conditions show that spatial patterns emerge near the positive constant solutions when the predator's intrinsic growth rate lies in a critical interval and the prey diffusion coefficient is smaller than the predator diffusion coefficient. Furthermore, the existence of bifurcating nonconstant steady states is rigorously established via the Lyapunov–Schmidt reduction. Numerical simulations confirm these analytical predictions and illustrate the spatiotemporal pattern formation process from disordered distributions to ordered patterns on both two-dimensional domains and torus surfaces. From an ecological perspective, the Turing instability results obtained here provide useful insights. The emergence of spatial patterns may serve as an indicator of local extinction risk or invasive species outbreaks. Moreover, the critical diffusion ratio identified by the Turing instability conditions can inform practical applications such as habitat corridor design and the establishment of isolation zones for invasive species. Moreover, it is worth pointing out that the current model can be extended in several directions. First, incorporating time delays to represent predator gestation periods or maturation time may reveal richer bifurcation phenomena. Second, considering stochastic perturbations or heterogeneous environments would help describe population dynamics in complex ecosystems more realistically. Third, extending the analysis to higher-dimensional spatial domains and exploring the influence of different boundary conditions remain worthwhile research directions.

## CRediT authorship contribution statement

**Ye Han:** Writing – review & editing, Writing – original draft, Validation, Investigation, Funding acquisition, Formal analysis. **Wanqin Wu:** Writing – review & editing, Supervision. **Xuewen Tan:** Writing – review & editing, Supervision, Formal analysis. **Xinzhi Liu:** Writing – review & editing, Formal analysis. **Wenjie Li:** Writing – review & editing, Supervision, Methodology, Formal analysis.

## Data availability

The data that support the findings of this study are available on request from the corresponding author, upon reasonable request.

## Declaration on conflicts of interest

The authors have no conflict of interest to declare in carrying out this research work.
